# Two inbred rat strains contrasting for anxiety-related behaviors show similar levels of defensive responses to cat odor

**DOI:** 10.1186/1744-9081-3-17

**Published:** 2007-04-13

**Authors:** Gustavo R Brüske, Leandro F Vendruscolo, André Ramos

**Affiliations:** 1 Laboratório de Genética do Comportamento, Departamento de Biologia Celular, Embriologia e Genética, Universidade Federal de Santa Catarina, Florianópolis, SC, Brazil

## Abstract

Rodents are known to display fear-related responses when exposed to the odor of natural predators, such as cats, even when they are totally naïve to these stimuli. Based on that, a behavioral test in which rats are exposed to cat odor has been developed and proposed to model some forms of anxiety. The objective of the present study was thus to compare the LEW (Lewis) and SHR (spontaneously hypertensive rats) inbred rat strains, which display genetic differences in other classical models of anxiety, in the cat odor test. As expected, cat odor produced an increase in fear-related behaviors. However, no clear differences were found between the two strains tested. These results suggest that the type of stress experienced by LEW and SHR strains exposed to cat odor is different from that elicited by exposure to classical models of anxiety such as the elevated plus-maze, black/white box and open-field tests.

## Background

Many studies have shown that laboratory rodents that have never been exposed to a live cat (or any cat vestiges), demonstrate strong fear responses when exposed to cat odor [1-13]. Pharmacological studies that use responses of rodents to cat odor as a model of human anxiety have produced controversial results. Benzodiazepine drugs, which are effective against generalized anxiety disorder in humans [14] and in classical animal models of anxiety (e.g. elevated plus-maze, black/white box, open-field) [6,8-10,15-19], can sometimes modulate the defensive responses of rodents to cat odor [2-4,8,9]. In other studies, however, benzodiazepines did not change the defensive behavior of rats [6,10] or mice [1,10] exposed to cat odor. Zangrossi and File [11] reported that chlordiazepoxide reduced anxiety evaluated in the social interaction and elevated plus-maze tests after exposure to cat odor, but it had only a limited effect on the direct responses of rats exposed to cat odor. These findings suggest that the cat odor test (COT), while sharing some common aspects with other recognized anxiety models, may be relevant to our understanding of some specific forms of anxiety in humans [5,11-13]. However, the type of emotionality measured in this test still requires elucidation. To our knowledge, no direct comparisons of rat strains contrasting for their emotionality levels have been carried out in the COT, which would shed some light on the psychological significance of this test.

The inbred rat strains Lewis (LEW) and Spontaneously Hypertensive Rats (SHR), when compared with each other, display high and low basal indices of anxiety-related behaviors, respectively, when tested in classical anxiety models. These strains, however, do not differ in their activity levels in either novel or familiar environments [18-25] but pharmacological studies indicate that they respond differently to the anxiolytic effects of benzodiazepines [18,19,21]. The anxiety-related differences between LEW and SHR were found to be due to genetic effects [20,22,24]. Therefore, this pair of rat strains provides a useful genetic model for the experimental study of anxiety.

In spite of showing consistent differences in a variety of anxiety models, LEW and SHR rats do not differ in the social interaction test of anxiety [21] and in their behavioral and physiological stress responses elicited by the exposure to fox odor [26]. Thus, the study of these strains in the COT should be useful to improve our psychological understanding of both the test and the rat strains. The objective of the present study was to compare the LEW and SHR strains in the COT, with or without the presence of the predator's odor. Animals of both sexes were included because there is considerable evidence for sex differences in emotional reactivity [20,24,25,27].

LEW and SHR rats (16/strain/sex) coming from our own colonies were used [22]. All animals were kept in collective plastic cages (5 rats/cage) with food and water available *ad libitum *under a 12-h light/dark cycle (lights on at 07:00 h) at 21 ± 2°C.

The apparatus was made of wood covered with formica and consisted of a rectangular box divided in two compartments: one smaller sheltered area covered with a ceiling (25 × 25 × 30 cm height) and one larger open area (40 × 25 × 30 cm height) where a collar, worn (cat odor) or not (control) by a cat during 3 weeks was hung in the opposite end in relation to the entrance (6 × 6 cm) of the shelter. The light inside the open area was at 7 lux. Each rat was placed in the center of the open area, facing the collar, and the following measures were registered for 5 min: the time spent with all four paws outside the shelter, the time spent in direct physical contact with the collar, the number of approaches towards the collar and the number of transitions between the sheltered and the open compartments. One transition was considered as each time that the animal left the shelter, went to the open area and came back to the shelter with all four paws. After experimental sessions the apparatus was cleaned with 70% ethanol. To minimize odor contamination, cat odor and control groups were tested in different days. Males and females were also tested in alternate days. For each sex, a total of four separate days were used. The animals were tested between 14:00 and 18:00 h.

The results were analyzed by a three-way ANOVA for the factors strain, sex and odor condition (cat odor vs. control). Newman-Keuls test was used for post-hoc comparisons. The accepted level of significance for all tests was p < 0.05.

**Figure 1:**
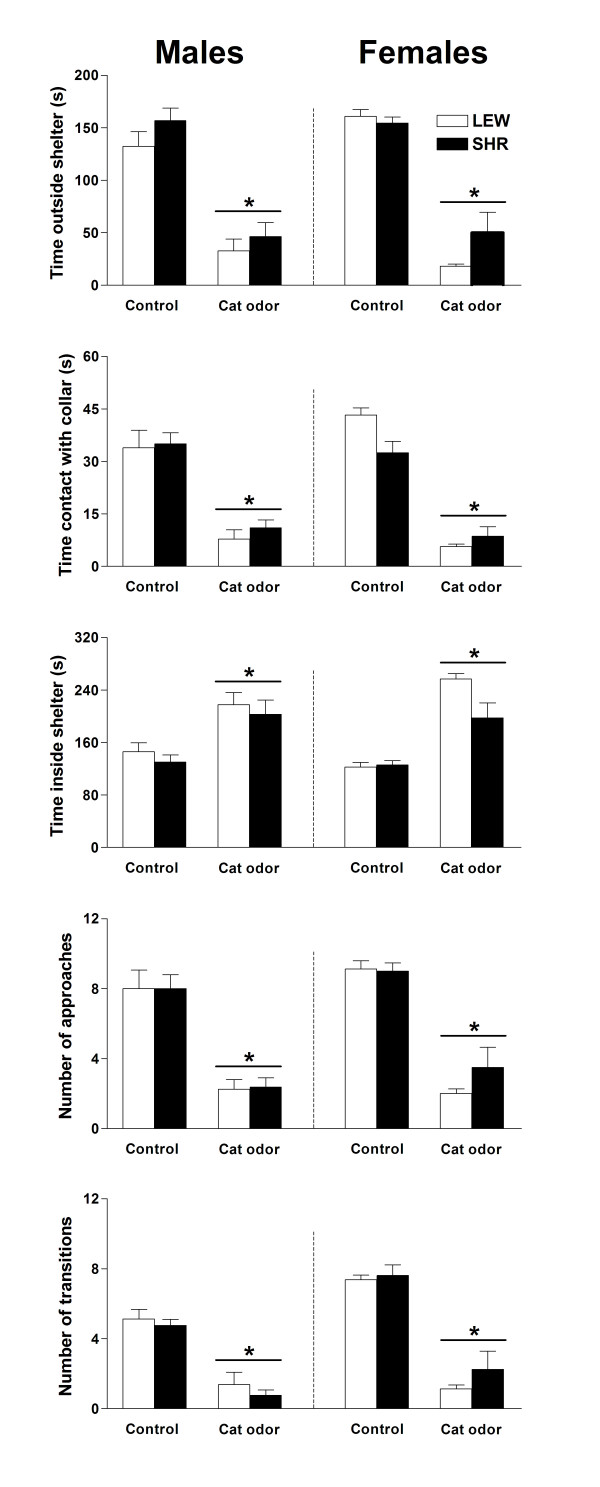
Time (s) spent outside the shelter, in contact with the collar, inside the shelter, and number of approaches and number of transitions for LEW and SHR rats of both sexes exposed to a collar with or without cat odor. The bars and vertical lines represent the means and SEM of animals grouped by strain and gender (n = 8). * Indicates overall odor effect (control vs. cat odor; ANOVA, p < 0.05).

The results of the behavioral responses showed by SHR and LEW rats of both sexes exposed to a collar with or without cat odor are illustrated in Figure 1. The three-way ANOVA revealed an overall effect of odor (cat odor vs. control) for all variables, with the animals exposed to the apparatus containing a collar with cat odor spending less time outside the shelter (F_(1,56) _= 191.2; p < 0.0001) and in contact with the collar (F_(1,56) _= 172.8; p < 0.0001), spending more time inside the shelter (F_(1,56) _= 70.1; p < 0.0001), approaching less frequently the collar (F_(1,56) _= 135.4; p < 0.0001) and making less transitions (F_(1,56) _= 146.1; p < 0.0001) between compartments than the animals exposed to the apparatus containing the collar alone (control). Moreover, there was an overall effect of strain for the time spent inside the shelter (F_(1,56) _= 4.5; p < 0.0375), with LEW rats spending more time inside this compartment than SHRs. Furthermore, an overall effect of sex (F_(1,56) _= 15.8; p < 0.0002) and an interaction between sex and odor (F_(1,56) _= 5.8; p < 0.0189) were detected for the number of transitions. The post-hoc comparisons indicated that males made less transitions under the control condition than females (p < 0.0002).

In agreement with the concept that emotionality is a multidimensional trait, studies applying multiple behavioral tests on groups of animals with well defined genotypes can be useful to investigate whether or not different experimental paradigms (or different testing conditions) assess the same psychological phenomenon [23]. In this study, the LEW and SHR strains, which are known to differ in several behavioral tests of anxiety/emotionality [18-25], displayed similar anxiety-like responses when evaluated in the COT. All groups highly avoided the collar and the environment containing the cat odor. However, some specific differences were observed: the LEW rats spent more time inside the small sheltered area, regardless of the odor condition, than SHRs, corroborating the more emotional profile of the former strain [18-25] and, males made fewer transitions under the control condition than females, thus confirming the well known sex differences in locomotion [20-25].

Compared to SHRs, LEW rats of both sexes display lower levels of approach towards the aversive, less-protected areas of the open-field, elevated plus-maze, and black/white box and show increased startle reflex, which suggests that they are more anxious-like than SHRs [18-25,28]. LEW rats submitted to some stressful situations show more severe and/or longer-lasting stress responses than SHRs [27,29]. In most but not all of these aforementioned tests, the females were less anxious-like than males. We have reported that LEW and SHR strains display similar levels of behavioral and neuroendocrine responses when exposed to fox odor [26]. This latter evidence together with the present results suggest that the type of emotional stress experienced in classical models of anxiety (and in some other stressful conditions) is different from that experienced in tests containing predator odors. Therefore, behavioral and physiological responses to these different types of environmental challenges are probably under control of different genetic mechanisms. The SHR strain, besides being used in the study of anxiety, also provides an important model of attention deficit hyperactivity disorder for showing hyperactivity, inattention and impulsiveness [30-32] when compared with other strains such as Wistar Kyoto. The influence of these characteristics on the so-called anxiety-related behaviors cannot be overlooked

The present results agreed with Hogg and File [7] who reported that rats differing in their reactivity to cat odor displayed similar anxiety levels in the elevated plus-maze and social interaction tests. However, gender differences related to anxiety/emotionality frequently observed in other anxiety models [20-25] were not clearly seen in the present study.

Zangrossi and File [11-13] have proposed that during exposure to cat odor the responses of rats have features of specific phobic anxiety, as benzodiazepines did not attenuate the defensive responses of the animals to this stimulus [6,10,11]. Conversely, other studies have shown reduction of these defensive responses by benzodiazepines [2-4,8,9]. Griebel et al. [5] have proposed that some defensive responses of rodents elicited by predators resemble panic attacks. The discrepancies across studies may result from differences in the type of measures, procedures, apparatuses, etc. Herein, we have used a model which is fairly similar to that proposed by Dielenberg and McGregor [3,4,8,9], in which benzodiazepines were effective.

In conclusion, the expected behavioral differences between the LEW and SHR strains, which differ in some classical models of generalized anxiety, were not found in the COT. Further evaluation of these rats, with and without pharmacological treatment, in tests with predator odors as well as in other behavioral tests, should improve our understanding of their psychological profile, which will represent an important step towards the investigation of new neurobiological/genetic mechanisms underlying anxiety-related traits.

## Competing interests

The author(s) declare that they have no competing interests.

## Authors' contributions

GRB carried out the data collection, helped to design the study and wrote the manuscript. LFV participated in the data analyses, interpretation of data and elaboration of the manuscript. AR designed the study and the data analysis strategy, and participated in the interpretation of data and elaboration of the manuscript. All authors read and approved the final manuscript.
